# Satyrization in *Drosophila* fruitflies

**DOI:** 10.1111/jeb.13733

**Published:** 2020-12-02

**Authors:** Stewart Leigh, Wayne G. Rostant, Martin I. Taylor, Luke Alphey, Tracey Chapman

**Affiliations:** ^1^ School of Biological Sciences University of East Anglia Norwich UK; ^2^ The Pirbright Institute Woking UK

**Keywords:** *Aedes*, *Drosophila*, hybrid mating, satyrization, seminal fluid proteins, speciation

## Abstract

The satyr of Greek mythology was half‐man, half‐goat, with an animal persona signifying immoderate sexual appetites. In biology, satyrization is the disruption of reproduction in matings between closely related species. Interestingly, its effects are often reciprocally asymmetric, manifesting more strongly in one direction of heterospecific mating than the other. Heterospecific matings are well known to result in female fitness costs due to the production of sterile or inviable hybrid offspring and can also occur due to reduced female sexual receptivity, lowering the likelihood of any subsequent conspecific matings. Here we investigated the costs and mechanisms of satyrization in the *Drosophila melanogaster* species subgroup of fruitflies. The results showed that *D. simulans* females experienced higher fitness costs from a loss of remating opportunities due to significantly reduced post‐mating sexual receptivity than did *D. melanogaster* females, as a result of reciprocal heterospecific matings. Reciprocal tests of the effects of male reproductive accessory gland protein (Acp) injections on female receptivity in pairwise comparisons between *D. melanogaster* and five other species within the *melanogaster* species subgroup revealed significant post‐mating receptivity asymmetries. This was due to variation in the effects of heterospecific Acps within species with which *D. melanogaster* can mate, and significant but nonasymmetric Acp effects in species with which it cannot. We conclude that asymmetric satyrization due to post‐mating effects of Acps may be common among diverging and hybridising species. The findings are of interest in understanding the evolution of reproductive isolation and species divergence.

## Graphical abstract

Seminal Fluid Proteins (Sfps) are transferred with the ejacuate during mating, conferring a variety of post‐mating effects on females. We found asymmetries in the strength and effects of these Sfps in reciprocal cross‐species comparisons between closely related *Drosophila* species.

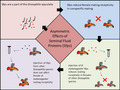

## 1 INTRODUCTION

Reproductive interference occurs when the courtship and copulation of one species is interrupted or disturbed by another (Gröning & Hochkirch, 2008). It has been observed across many taxa (de Bruyn et al., 2008; Landolt & Heath, 1987; Seehausen et al., 1997; Shuker & Burdfield‐Steel, 2017) and can take many forms, including signal blocking, heterospecific rivalry and heterospecific mating (Gröning & Hochkirch, 2008). In insects and other animals, reproductive interference is often referred to as satyrization (Ribeiro & Spielman, 1986). The effects of satyrization can be symmetric or asymmetric, depending on the frequency of heterospecific mating, degree of reproductive incompatibility and strength of post‐mating effects. Asymmetric satyrization influences the level of interspecific competition between species that hybrid mate, with greater asymmetry increasing the probability of competitive exclusion (Kishi & Nakazawa, 2013). This is an important consequence of heterospecific mating and is of interest in understanding reinforcement and species divergence (Matute, 2010) as well as in practical applications of satyrization as a method of insect control (Kishi & Nakazawa, 2013). Satyrization can occur before and after mating. Asymmetries in premating satyrization costs arise when the probability of reciprocal heterospecific matings differs, due to divergent and incomplete mate recognition barriers, facilitating heterospecific mating in one direction at higher frequency than the other. Fitness effects primarily arise as opportunity for remating, energetic or mating trauma costs (Yassin & David, 2016).

Heterospecific matings are well known to result in the production of infertile or inviable hybrid offspring (Coyne & Orr, 1989, 1997; Turissini et al., 2018). They can also result in the inhibition of sexual receptivity in heterospecific females, leading to fewer rematings with conspecific males. Seminal fluid proteins (Sfps) govern the extent to which heterospecifically mated females increase their egg production, decrease their subsequent receptivity and store or release sperm (Chapman, 2001; Rubinstein & Wolfner, 2013; Sepil et al., 2019; Sirot et al., 2014). As such, Sfps, including their major constituents, the accessory gland proteins (Acps), are predicted to be key determinants of the magnitude and asymmetry of post‐mating satyrization effects. Sfps represent a diverse cocktail of proteins that form the nonsperm part of the male ejaculate of most species of insects and other animals. There are >200 Sfps in *D. melanogaster* (Mueller et al., 2005; Findlay et al., 2008; Findlay et al., 2009; Sirot, LaFlamme, et al., 2009; Sepil et al., 2019) that influence many post‐mating behavioural and physiological responses, such as ovulation, sperm storage and mating receptivity (Chapman et al., 2003; Chapman & Davies, 2004; Hollis et al., 2019; Liu & Kubli, 2003; Rubinstein & Wolfner, 2013).

Approximately 10% of the genes encoding Sfps evolve rapidly (Haerty et al., 2007; Mueller et al., 2005; Swanson & Vacquier, 2002). Though many *D. melanogaster* Sfps are orthologous to those found in other species within the *Drosophila melanogaster* species subgroup, others are species‐specific (Findlay et al., 2008). As a result of this rapid evolution, Sfps may quickly become incompatible across diverging species, facilitating reproductive isolation (Andrés et al., 2008; van Doorn et al., 2009; Goenaga et al., 2015). Therefore, Sfps are expected to have variable heterospecific effects (Dapper & Wade, 2016; Tsuda & Aigaki, 2016) and could contribute to significant post‐mating satyrization. Lineage‐specific differences in the rate of evolutionary change of Sfps vs. their receptors in females could generate significant asymmetries indicative of satyrization (Ahmed‐Braimah et al., 2017). Sfps with functional effects in the heterospecific context would render females refractory to further matings with conspecifics and induce costs in terms of ‘time out’ of the mating pool and through the production of infertile or sterile offspring.

Reproductive incompatibilities may also be impacted and potentially ameliorated, by conspecific sperm precedence (Castillo & Moyle, 2019; Manier, Belote, et al., 2013; Manier, Lüpold, et al., 2013; Price, 1997; Turissini et al., 2018). Several species within the *D. melanogaster* species subgroup exhibit conspecific sperm precedence, that is, in situations in which females are carrying sperm from both conspecific and heterospecific males, conspecific sperm will be preferentially used to fertilize eggs. While this phenomenon may reduce costs of satyrization through lower production of infertile/sterile hybrid offspring, it does not reduce conspecific mating opportunities lost to heterospecific matings, which are predicted to be significant and contribute to competitive exclusion (Noriyuki et al., 2012). Such costs are predicted to lead to selection for reinforcement to avoid such heterospecific matings (Matute, 2010).

As yet, neither the frequency of asymmetric satyrization, nor the post‐mating mechanisms underlying it, are fully resolved. Potential markers of satyrization include differences in incomplete mate recognition and Sfps that show variable functional effects in heterospecific mating. Both of these effects are reported in natural populations of *Aedes* mosquitoes, which are vectors of harmful diseases such as Dengue, Zika and Yellow Fever (Alto et al., 2014; Hugo et al., 2019; Johnson et al., 2002). *Ae. aegypti* females will readily mate with *Ae. albopictus* males, whereas the reciprocal mating does not occur. Hence, *Ae. aegypti* females frequently receive Sfps from *Ae. albopictus* males, causing an increase in the production of infertile eggs and rendering *Ae. aegypti* females less willing to mate with conspecifics. Therefore, *Ae. aegypti* (but not *Ae. albopictus*) females can suffer significant costs from asymmetric satyrization. This is thought to be a major contributor to the observation that *Ae. albopictus* replaces *Ae. aegypti* via competitive exclusion in areas of sympatry (Tripet et al., 2011). *Ae. albopictus* is a less competent vector of Dengue, Zika and Yellow Fever than *Ae. aegypti* (Alto et al., 2014; Hugo et al., 2019; Johnson et al., 2002). Therefore, in this context, satyrization is of interest for insect control.

There is much interest in the relative contribution of premating and post‐mating processes to divergence in sympatry vs. allopatry (Matute, 2010). The underlying processes involved include those that lead to heterospecific matings (Turissini et al., 2018), the actions of Sfps (Sepil et al., 2019) and the relative rates of divergence of reproductive genes (Hollis et al., 2019). Overall, it is increasingly realized that post‐mating prezygotic processes can play an important role in initiating and driving reproductive isolation in all settings (Matute, 2010). Here, we build upon this recent interest by investigating these mechanisms in the context of satyrization. We investigated satyrization costs and mechanisms in experimentally tractable *Drosophila* fruiflies, with a primary focus on the effects of Acps. Our aim was to test the hypothesis that there are significant costs due to asymmetric satyrization, explore whether satyrization is asymmetric across a group of closely related species, and examine the role of Acps in this phenomenon. Previous work investigating satyrization in *Drosophila* has demonstrated that conspecific mating costs, in the form of physical trauma, are often amplified in heterospecific matings (Yassin & David, 2016). There is also is an extensive body of research into heterospecific matings specifically between *D. melanogaster* and *D. simulans* (e.g. Coyne & Orr, 1989, 1997). All hybrid progeny from *D. melanogaster* x *D. simulans* matings are sterile or infertile with differences in the frequency and consequences of reciprocal hybridizations reported.

We first tested for asymmetries in the frequency and post‐mating satyrization effects of reciprocal heterospecific matings between *D. melanogaster* and *D. simulans*, to estimate satyrization under our experimental conditions. We then tested for asymmetric satyrization in post‐mating responses across the *D. melanogaster* species subgroup. To do this, we documented female receptivity to mating after injections of conspecific or heterospecific Acps, vs. a saline control, in comparisons between *D. melanogaster* and five other members of the *D. melanogaster* species subgroup (Obbard et al., 2012). We used the frequency of copulations as a metric for sexual receptivity, measuring the difference in the number of copulations and speed of copulation onset between treatments. As satyrization includes both a premating and post‐mating component, we included three species with which *D. melanogaster* can physically copulate (*D. simulans, D. sechellia, D. teissieri*) and two with which it cannot (*D. erecta* and *D. yakuba*) (Turissini et al., 2018). ‘Post‐mating’ here refers to the inducement of physiological changes through the effect of Acps by injection into the abdomen, in the absence of actual mating. This allowed us to demonstrate the strength of post‐mating satyrization and test whether asymmetry in post‐mating satyrization is restricted to species that exhibit complete premating barriers which prevent heterospecific mating.

## 2 MATERIALS AND METHODS

### 2.1 Fly culturing and collection

Unless stated otherwise, *Drosophila* eggs were collected by placing a red grape juice agar plate (275 ml H_2_O, 12.5 g agar, 250 ml red grape juice, 10.5 ml 10% w/v Nipagin solution) into population cages containing the appropriate species. *D. melanogaster* was cultured in population cages containing overlapping generations at 25°C and 60% RH on a 12 hr:12 hr light:dark cycle. The cages hold 12 bottles each containing 70 ml of sugar yeast agar (SYA) medium (30 ml 10% w/v Nipagin solution, 3 ml propionic acid, 15 g agar, 50 g sugar and 100g brewer's yeast per litre), with the oldest three bottles being replaced each week. All other species (*D. simulans*, *D. yakuba*, *D. teissieri*, *D. erecta* and *D. sechellia*) were kept in SYA bottles under overlapping generations, cultured inside a 22°C incubator on a 12 hr:12 hr light:dark cycle and transferred to new SYA bottles every 2 weeks. All flies used in experiments were raised from egg to adult inside a constant temperature (CT) room at 25°C and 60%RH on a 12 hr:12 hr light:dark cycle unless specified otherwise. Egg collection plates were left in the cages for 3 hr, removed and then incubated. After 24 hr, first‐instar larvae of each species were picked from the plates and placed 100 per vial (75 x 25 mm), each containing 7 ml SYA. This procedure standardized the larval development across and within species and minimized any environmentally induced variation in body size. Virgin adult females and males were collected using ice anaesthesia and separated by sex. The sex‐segregated flies were then stored, 10 per vial for 3–6 days until their use in experiments.

### 2.2 Frequency of heterospecific and conspecific matings between *D. melanogaster* and *D. simulans*

Adult *D. melanogaster* (Dahomey) and *D. simulans* (National *Drosophila* Species Stock Center (DSSC)) wildtype flies were allocated at random to one of the four following experimental treatments: *D. simulans* (♀) x *D. simulans* (♂) *n* = 40; *D. melanogaster* (♀) x *D. melanogaster* (♂) *n* = 40; *D. simulans* (♀) x *D. melanogaster* (♂) *n* = 39*; D. melanogaster* (♀) x *D. simulans* (♂) *n* = 40 (Experiment 1A, Figure S1). One male and one female from each species were gently aspirated into a vial within 2 hr after lights on and were continuously observed for 3 hr, during which spot checks were also performed every 20 min to score courtship and copulation frequency. The mating duration of *D. melanogaster* pairs is approximately 15–20 min (Pavković‐Lučić et al., 2014). Hence behavioural spot checks captured all matings in the 3 hr spot check period without double counting them. The spot checks of behaviour were then repeated for the same 3 hr over the following 2 days.

### 2.3 Effects of hetero‐ and conspecific matings on female remating receptivity in *D. melanogaster* and *D. simulans*

*D. melanogaster* and *D. simulans* were collected as stated above and adults each aspirated into a vial with a conspecific or heterospecific male that had been placed in the vial 24 hr earlier (Experiment 1B, Figure S1). At 9:00 on the first day, pairs were continuously observed for 3 hr and mating latency and mating duration were recorded. After matings ended, males were immediately removed, and females retained in their vials for 24 hr. Unmated females were discarded. At 13:00 the next day, 24 hr after the previously mated females had finished mating, the females were transferred into a new vial containing a conspecific male and were observed for 3 hr to test for post‐mating receptivity. As before, mating latency and mating duration were recorded. No matings were observed between *D. melanogaster* (♀) x *D. simulans* (♂). Therefore, no females from this treatment were available for remating tests. Excess heterospecific pairs were set up to ensure sufficient mated females for rematings. The sample size set up for each treatment in each experiment and the number and percentage of pairs that mated are given in Table S1.

### 2.4 Effects of reciprocal Acp injections between *D. melanogaster* and 5 species of the *melanogaster* species subgroup

*D. melanogaster* (Dahomey) wild type was used in each experiment as the baseline against which to test wildtype flies of other members of the *D. melanogaster* species subgroup (Experiment 2, Figure S2). Each experiment consisted of saline, conspecific Acp and heterospecific Acp injections between *D. melanogaster* and another species – *D. sechellia* (KYORIN‐Fly Stock No. k‐s10), *D. simulans* (DSSC), *D. erecta* (K‐F Stock No. k‐s02), *D. teissieri* (DSSC) and *D. yakuba* (K‐F Stock No. k‐s03). These species are representatives from the two major clades of the *melanogaster* species subgroup and included three species with which *D. melanogaster* can heterospecifically mate (*D. sechellia*, *D. simulans* and *D. teissieri*) and two with which it cannot (*D. yakuba* and *D. erecta*) (Turissini et al., 2018).

To generate Sfp‐mediated post‐mating physiological effects, Acps were injected into females of each species. Acps were extracted from the entirety of the accessory gland, but did not include proteins from the ejaculatory duct (see dissection details, below). Male Acp donors, for tests with *D. melanogaster* x *D. simulans*/ *D. erecta*/ *D. yakuba* males, were collected within 24 hr of eclosion to standardize male age and stored 10 per vial for at least 48 hr to replenish Acps. Thus, the extracted Acps were from fully rested, sexually mature males, and of comparable status and volume across the different species tested. In tests with *D. melanogaster* x *D. teissieri*/ *D. sechellia,* it was found that *D. teissieri* and *D. sechellia* showed low fecundity on egg collection plates and suffered high mortality at 25°C. Therefore, flies for these two experiments were cultivated in food vials for 8 hr and 16 hr laying periods at 22°C under 12 hr:12 hr light:dark cycle, 60% RH. Egg‐laying vials were set up, each containing 8 females and two males of the respective species (and 4 females and 1 male for *D. melanogaster* to control egg density across species). Adults were first placed into vials for an 8 hr egg‐laying period and then immediately transferred to new vials for 16 hr to lay eggs. Adult flies were removed after the egg‐laying period and the eggs from both oviposition collections placed at 22°C to develop to adult emergence, after which the males were collected and kept in single sex groups of 10 males for at least 48 hr to replenish Acps.

To prepare Acps for injection into females, 90–120 pairs of accessory glands were dissected from 2‐ to 4‐day‐old males of each species, separated from the ejaculatory duct, and placed into a microcentrifuge tube containing 1xPBS (phosphate‐buffered saline) at a concentration of 3 accessory gland pairs/μl of 1xPBS. These were stored at −20°C. The day before the injection experiment, the accessory gland pairs were sonicated in 1xPBS with 5x one second pulses and centrifuged at 12,000 g for 15 min at 4°C. The supernatant was placed into a new microcentrifuge tube and stored at −20°C.

Virgin females for injection were collected in the same way as the Acp donor males for each respective species and given 2–6 days to sexually mature before injection. On the day of the injection experiments, virgin females were anaesthetized on CO_2_ and injected with 0.1 μl of either 1xPBS, 0.1 μl of conspecific Acps or 0.1μl of heterospecific Acps. Acps were injected directly into the abdomen of each female (Tsuda & Aigaki, 2016). The volume of fluid injected represents 0.3–0.5 of an accessory gland equivalent and is comparable to the amount of Sfps received in a normal mating (Sirot et al., 2009). Immediately after injections, each female was placed into a separate vial containing yeast paste (to promote mating) and placed at 25°C (for experiments using *D. simulans*, *D. yakuba* and *D. erecta*) or 22°C (for experiments using *D. sechellia* and *D. teissieri*) for 24 hr. Eighty females per treatment were initially injected in each experiment to ensure a sufficient sample size for the subsequent mating assay (Table S2). Twenty‐four hours post‐injection, a conspecific male was placed into each vial containing a surviving female. Pairs were observed for 3 hr (4 hr for the *D. melanogaster* x *D. sechellia*/ *D. teissieri* experiments conducted at 22°C). Introduction of the male, mating start and mating finish times were recorded to assess the number of matings, mating latency and mating duration.

### 2.5 Statistical analysis

Copulation frequency and mating latency data were analysed by performing a Kruskal–Wallis test followed by Dunn's post hoc analysis to test for significant differences between treatments. Differences in the number of matings and rematings, and in post‐Acp injection survival, were analysed used a chi‐square test. Differences in female mating receptivity following Acp injection were analysed using a Cox proportional hazards model. A generalized linear model (GLM) was used to test for interaction effects between injection treatments and species of the injected female, with significant differences in the effects of the reciprocal Sfps being indicative of satyrization asymmetry. All analyses were carried out in R v3.2.2 (R Core Team, 2012).

## 3 RESULTS

### 3.1 Frequency of hetero‐ and conspecific matings between ****D*** .  ***melanogaster* and *D*** .  ***simulans****

**Figure 1:**
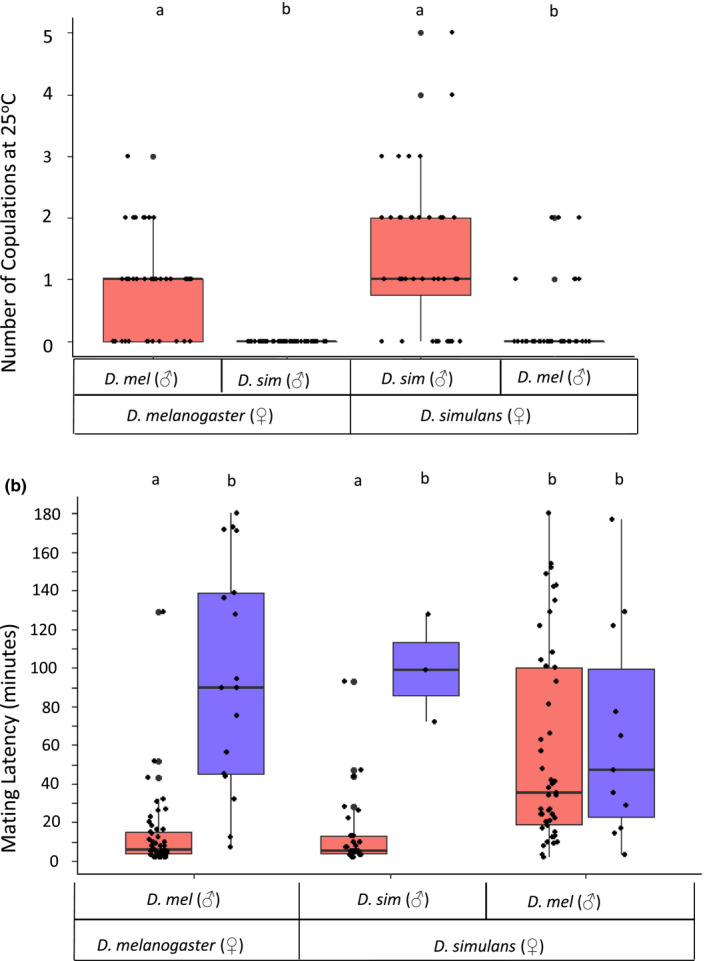
(a) Conspecific and heterospecific matings observed between *D. melanogaster* and *D. simulans*, tested at 25°C. Observations of mating behaviour were conducted every 20 min for 3 hr after lights on over three consecutive days. Sample sizes are *D. simulans* (♀) x *D. melanogaster* (♂) *n* = 39; *D. melanogaster* (♀) x *D. simulans* (♂) *n* = 40; *D. melanogaster* (♀) x *D. melanogaster* (♂) *n* = 40; *D. simulans* (♀) x *D. simulans* (♂) *n* = 40. (b) Mating latency (mins) during the first (red) and second (blue) matings between *D. melanogaster* and *D. simulans,* tested at 25°C. X‐axis labels describe the treatments in the first mating. All mated females from the first mating were mated with a conspecific male for the second mating regardless of the species of the male from the first mating. The sample size set up for each treatment and the number and percentage that mated is shown in Table S2. Box plots show the median, 25%–75% IQ range, whiskers (1.5 x IQR) and outliers. Different letters indicate statistically significant differences between groups (*p* < .05)

Conspecific mating was significantly more frequent than heterospecific mating (Kruskal–Wallis H_(1)_ = 62.33; *p* = 2.911e‐15; Figure 1a) (Experiment 1A, Figure S1). Heterospecific matings between *D. melanogaster* and *D. simulans* were unidirectional, with approximately 33% of *D. simulans* females hybrid mating with *D. melanogaster* males, and no matings in the reciprocal direction (Figure 1a, Table S1).

### 3.2 Effects of hetero‐ and conspecific matings on female remating receptivity in *D. melanogaster* and *D. simulans*

During the first mating, conspecific pairs mated significantly more frequently when compared to heterospecific pairs ($\chi_{3}^{2}$ = 146.04, *p* = 2.2e‐16) and heterospecific mating was highly asymmetric, with matings occurring only between *D. simulans* (♀) x *D. melanogaster* (♂). Additionally, *D. simulans* (♀) x *D. melanogaster* (♂) took significantly longer to start mating (H_2_ = 42.22; *p* = 6.811e‐10) than the two conspecific treatments (Figure 1b) (Experiment 1B, Figure S1). During the second mating, when all females were paired with a conspecific male, all three treatments had a relatively low remating rate with no significant difference between them ($\chi_{2}^{2}$ = 5.63, *p* = .06). There were also no significant differences in mating latency between any of the treatments (H_2_ = 2.38; *p* = .305), demonstrating that the post‐mating refractory effect induced by *D. melanogaster* males was similar in conspecific *D. melanogaster* and heterospecific *D. simulans* females. Hence, heterospecifically mated *D. simulans* females showed a significantly reduced propensity to remate, leading to a potentially costly period of elevated production of sterile or inviable offspring production. As the heterospecific matings were unidirectional, only *D. simulans* incurred this post‐mating cost.

### 3.3 Effects of reciprocal Acp receipt across the *melanogaster* species subgroup

**Figure 2:**
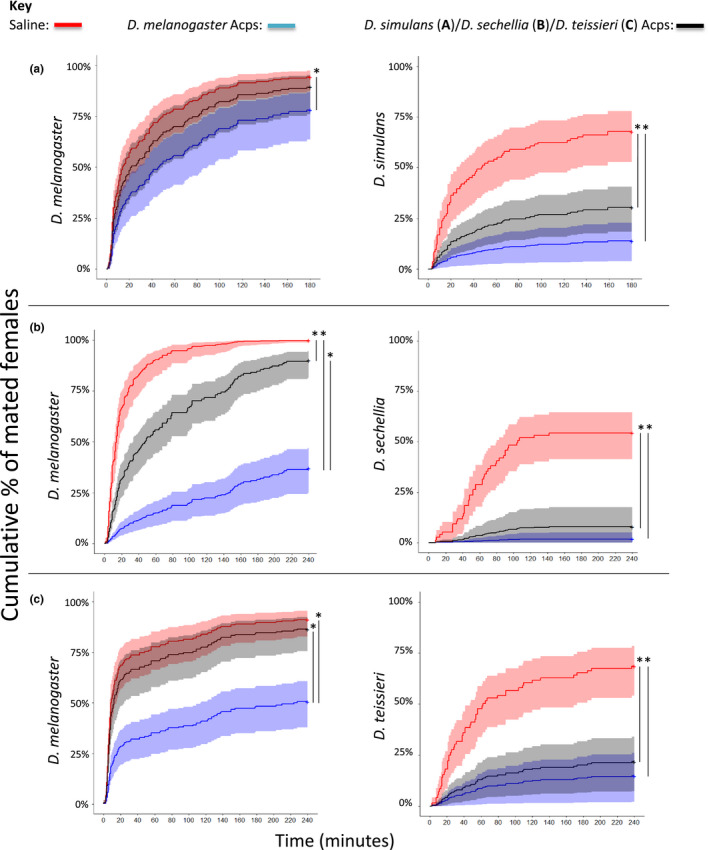
Asymmetrical post‐mating responses between members of the *D. melanogaster* species subgroup. Shown is the Cox proportional hazards model of females that mated over the 3 hr mating assay period, 24 hr following injection with either saline (red), *D. melanogaster*Acps (blue) or *D. simulans* (a), *D. sechellia* (b) and *D. teissieri* (c) Acps (black). Asymmetry is revealed by a comparison of the left and right panels. Shown in the shaded areas are the 95% confidence intervals for each treatment, asterisks indicate significant differences between treatments connected by black lines (*p* < .05). Sample sizes are – *D. melanogaster* and *D. simulans*: Saline x *D. mel* ♀ = 69, *D. mel* Sfps x *D. mel* ♀ = 44*, D. sim*Acps x *D. mel* ♀ = 36, Saline x *D. sim* ♀ = 54, *D. mel*Acps x *D. sim* ♀ = 50, *D. sim* Sfps x *D. sim* ♀ = 65, *D. melanogaster* and *D. sechellia*: Saline x *D. mel* ♀ = 74, *D. mel*Acps x *D. mel* ♀ = 71, *D. sec*Acps x *D. mel* ♀ = 74, Saline x *D. sec* ♀ = 63, *D. mel*Acps x *D. sec* ♀ = 58, *D. sec*Acps x *D. sec* ♀ = 25; *D. melanogaster* and *D. teissieri*: Saline x *D. mel* ♀ = 69, *D. mel*Acps x *D. mel* ♀ = 66, *D. tei*Acps x *D. mel* ♀ = 60, Saline x *D. tei* ♀ = 58, *D. mel*Acps x *D. tei* ♀ = 33, *D. tei*Acps x *D. tei* ♀ = 36

**Figure 3:**
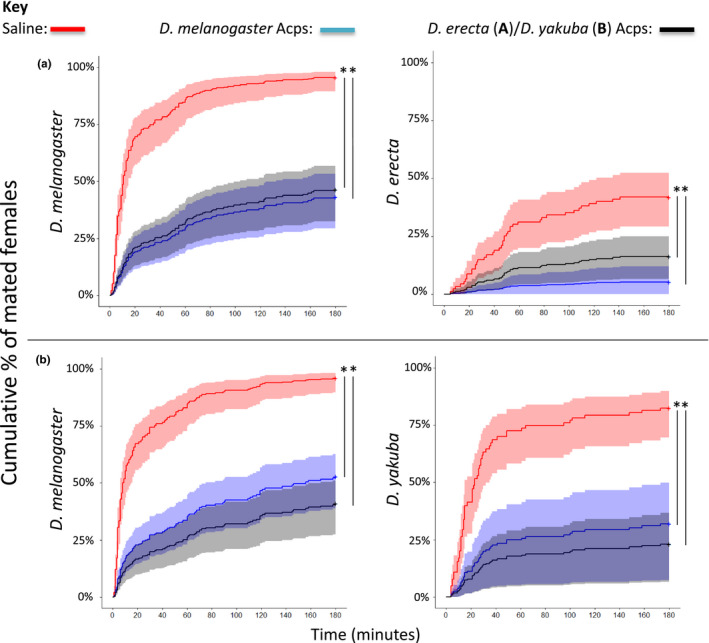
Symmetrical post‐mating responses between members of the *D. melanogaster* species subgroup. Shown is the Cox proportional hazards model of females that mated over the 3 hr mating assay period, 24 hr following injection with either saline (red), *D. melanogaster*Acps (blue) or *D. erecta* (a), and *D. yakuba* (b) Acps (black). Shown in the shaded areas are the 95% confidence intervals for each treatment, asterisks indicate significant differences between treatments connected by black lines (*p* < .05). Sample sizes are – *D. melanogaster* and *D. erecta*: Saline x *D. mel* ♀ = 72, *D. mel*Acps x *D. mel* ♀ = 62, *D. ere*Acps x *D. mel* ♀ = 62, Saline x *D. ere* ♀ = 67, *D. mel*Acps x *D. ere* ♀ = 38, *D. ere*Acps x *D. ere* ♀ = 64; *D. melanogaster* and *D. yakuba*: Saline x *D. mel* ♀ = 71, *D. mel*Acps x *D. mel* ♀ = 66, *D. yak*Acps x *D. mel* ♀ = 64, Saline x *D. yak* ♀ = 55, *D. mel*Acps x *D. yak* ♀ = 18, *D. yak*Acps x *D. yak* ♀ = 30

Overall, significant asymmetries in female receptivity were seen following reciprocal Acp injections in comparisons between *D. melanogaster* and *D. simulans, D. sechellia* and *D. teissieri* but not between *D. melanogaster* and *D. erecta* and *D. yakuba*. *D. melanogaster* Acps significantly reduced mating receptivity in *D. simulans*, *D. sechellia* and *D. teissieri* females (Experiment 2, Figure S2). However, the Acps from these three species either had no, or a significantly weaker, effect than *D. melanogaster* Acps on receptivity in the reciprocal tests in *D. melanogaster* females (Figure 2). In contrast, no significant asymmetries in female receptivity were seen in reciprocal Acp injections between *D. melanogaster* and *D. erecta* or *D. yakuba* (Figure 3). In these species, the Acps significantly reduced female receptivity equally in conspecific and heterospecific comparisons. Asymmetries in pairwise Sfp injections was supported by the GLM analyses, which showed significant interaction effects in many species, whereby the degree to which Acps were effective in reducing mating latency were dependent on both the substance injected into the female and the species of injected female (significant interaction effects: between *D. melanogaster* and *D. simulans* *F*_(2,312)_ = 4.74; *p* = .009, between *D. melanogaster* and *D. sechellia* *F*_(2,361)_ = 15.83; *p* = 2.6e‐07, between *D. melanogaster* and *D. teissieri* *F*_(2,316)_ = 7.31; *p* = 7.89e‐04, between *D. melanogaster* and *D. erecta* *F*_(2,359)_ = 8.99; *p* = 1.546e‐04). The *D. melanogaster* and *D. yakuba* comparison was the exception to this, showing no significant interaction effect (*F*_(2,298)_ = 0.2; *p* = .816) (see Supporting information for results of full analyses).

### 3.4 Effects of reciprocal Acp receipt on female survival across the *melanogaster* species subgroup

The number of females surviving following the Acp injections varied widely (Table S2) (saline: 67%–93%; conspecific Acps: 38%–89%; heterospecific Acps 23%–93%) (Experiment 2, Figure S2). In general, saline injections were less harmful to female survival than either con‐ or heterospecific Acp injections. *D. melanogaster* females were resistant to most injections of conspecific and heterospecific Acps with no significant differences between Acp and saline injections in any of the injection experiments except for *D. melanogaster* x *D. simulans*, where there was significantly lower mortality following saline injections compared to both con‐ and heterospecific Acps ($\chi_{2}^{2}$ = 33.25; *p* = 6.016e‐08). *D. yakuba* and *D. teissieri* were particularly sensitive to Acp injections, with females suffering significantly higher mortality when injected with Acps from both con‐ and heterospecific Acps compared to the saline control (*D. yakuba:* $\chi_{2}^{2}$ = 39.37; *p* = 2.824e‐9. *D. teissieri*: $\chi_{2}^{2}$ = 20.32; *p* = 3.862e‐05) (see Supporting information for a full breakdown of injection mortality).

## 4 DISCUSSION

Our results show significant costs of satyrization for *D. simulans* females that mated with *D. melanogaster* males, which were not observed in the reciprocal cross. *D. simulans* females mated at a reasonable frequency with *D. melanogaster* males, producing offspring with zero fitness, and showed significant reluctance to remate. In a natural setting, this may result in the female spending a significant time out of the mating pool – though any costs would be tempered by conspecific sperm precedence (Price, 1997). We examined the contribution of post‐mating effects to satyrization, by using Acp injection assays. This showed that Acps from all five species tested significantly reduced subsequent sexual receptivity in their own species in comparison with the saline control. Acps from *D. melanogaster* significantly reduced heterospecific female receptivity in all five species to the same extent as each of the five species own conspecific Acps. However, there were asymmetries in the degree to which Acps from other species were active in *D. melanogaster* females. Acps from *D. simulans*, *D. teissieri* and *D. sechellia* (with which *D. melanogaster* can naturally hybridize) had either no, or reduced effect on subsequent *D. melanogaster* receptivity. In contrast, Acps from *D. erecta* and *D. yakuba* (with which *D. melanogaster* does not hybridize) were just as effective as conspecific Acps in reducing female receptivity.

Stronger asymmetries in the fitness effects of heterospecific matings can facilitate competitive exclusion between two species (Kishi & Nakazawa, 2013). The frequency of heterospecific matings can play a significant role in this process (Matute, 2010). Our results supported the extensive previous evidence for asymmetric premating satyrization between *D. melanogaster* and *D. simulans* (Barbash, 2010; Barker, 1962; Coyne & Orr, 1989, 1997; Moulin et al., 2004; Sperlich, 1962; Sturtevant, 1920; Turissini et al., 2018). Heterospecific matings occurred unidirectionally, with *D. melanogaster* males mating infrequently with *D. simulans* females but with the reciprocal cross occurring at zero frequency. Therefore, *D. simulans* females that mated with *D. melanogaster* males incurred significant fitness costs in terms of the production of inviable or sterile hybrid offspring (Barbash, 2010) and reduced willingness to remate with conspecifics and thus receive conspecific sperm. Conspecific matings were significantly more frequent and were shorter to initiate than heterospecific matings between *D. melanogaster* and *D. simulans*. This is consistent with reports that incomplete mate recognition contributes to hybridizations between these species and suggests mate recognition control by females (Barbash, 2010). Almost all conspecific pairs mated and some pairs mated several times. *D. simulans* (♀) x *D. melanogaster* (♂) pairs mated more frequently than the reciprocal cross which was not observed at all in the mating tests performed here. However, even the most frequent heterospecific matings only occurred at about a third as often as for conspecifics. This provides evidence for premating satyrization – in addition, the presence of unidirectional heterospecific mating (and associated post‐mating effects described below) resulted in females of only one species suffering fitness costs of heterospecific mating. Some previous studies have observed that heterospecific matings between *D. melanogaster* females and *D. simulans* males are more frequent than the reciprocal (Moulin et al., 2004; Sperlich, 1962; Sturtevant, 1920). Our results contrast with this observation, but are in agreement with other reports of exclusive, unidirectional heterospecific mating between *D. melanogaster* males and *D. simulans* females (Barker, 1962). The pattern of unidirectionality in matings between *D. melanogaster* x *D. simulans* thus appears to be strain dependent and should be investigated in future work.

Because heterospecifically mated females in species pairs in which heterospecific Acps are active refrain, at least temporarily, from remating with conspecific males, satyrization should be most costly to the species in which females show greater receptivity to initial heterospecific matings. Here, there was no significant difference in remating behaviour between *D. simulans* females that mated first with either *D. melanogaster* or *D. simulans* males. Therefore, *D. simulans* females incurred costs from the receipt of heterospecific Acps, as prior mating to *D. melanogaster* males caused them to be less receptive to further mating. The effect of *D. melanogaster* Acps on *D. simulans* females is evidence for post‐mating asymmetric satyrization.

The results suggest that, in addition to any direct ecological competition when in sympatry, either of *D. melanogaster* or *D. simulans* could be at a potential disadvantage from asymmetric satyrization effects. This is dependent upon the direction of asymmetry which varies across different strains, at least in terms of premating effects (Barker, 1962; Moulin et al., 2004; Sperlich, 1962; Sturtevant, 1920)). Costs of satyrization will be diminished if there is strong conspecific sperm precedence (Castillo & Moyle, 2019; Manier, Belote, et al., 2013; Manier, Lüpold, et al., 2013; Price, 1997; Turissini et al., 2018). However, the effects of satyrization could also show density dependence. For example, at high‐density *D. simulans* females might more rapidly find *D. simulan*s males (or *vice versa*) and mate, whereas at low density, especially low‐*D. simulans* high‐*D. melanogaster*, the *D. simulans* females might only ‘see’ *D. melanogaster* males and suffer proportionately higher costs of satyrization. Future experiments and modelling to explore the potential for such density dependence would be useful.

Interestingly, we observed that post‐mating asymmetries were prevalent within the *melanogaster* species subgroup (Yassin & David, 2016). Asymmetries in post‐mating receptivity responses were seen between *D. melanogaster* and *D. simulans, D. sechellia* and *D. teissieri*. In each case, *D. melanogaster* Sfps significantly reduced receptivity in females of the reciprocal species, but the reciprocal species Acps produced either no significant effect or a significantly weaker effect when injected into *D. melanogaster* females. There was no asymmetry in the injections between *D. melanogaster* and *D. erecta* or *D. yakuba*. In these tests, all Sfps from conspecific or heterospecific species significantly reduced mating receptivity to the same extent.

Female mortality following Acp injections varied across species, with *D. melanogaster* suffering low mortality from most Acp injections, but *D. yakuba* and *D. teissieri* being particularly sensitive. High mortality may have been an artefact of the experiment itself. Injections may be traumatic, causing wounding and introducing into the female's body cavity a foreign substance. Interestingly, saline injections either showed no significant difference or were less harmful to females than receipt of con‐ or heterospecific Acps. This suggested that factors aside from the physical trauma associated with injection may have been having an effect. Nonsterile nonself material entering the female may have resulted in infection. Infection may have resulted in female mortality or prompted an immune response which may also have induced mortality costs. Some species suffered high mortality from only conspecific Acps (*D. sechellia*), some from only heterospecific Acps (*D. erecta*), and some from both (*D. teissieri, D. yakuba*). It would be interesting to investigate this in more depth.

Overall, asymmetry in post‐mating effects was found only in different species which can engage in heterospecific mating (Turissini et al., 2018) suggesting that asymmetries occurred between species that are more closely related (Balakrishnan et al., 2009; Miller et al., 2019; Moulin et al., 2004; Sato et al., 2015; Schwarz & McPheron, 2007). *D. yakuba* and *D. erecta* are more phylogenetically distant to *D. melanogaster* than are *D. simulans* and *D. sechellia*, although *D. teissieri* seems to lie between *D. erecta* and *D. yakuba* (Obbard et al., 2012). That asymmetric satyrization occurred in all of the most closely related members tested could suggest that it is widespread. In areas in which closely related species have overlapping ranges, satyrization could shape interactions between closely related sympatric species.

Why there might be a link between the ability to hybridize and asymmetrical post‐mating effects of Acps is not yet known, but two possibilities are described below:

1. *Evolution of resistance to costly heterospecific matings*. Diverged species have generally evolved complete premating barriers which can take the form of behavioural or mechanical premating isolation mechanisms (Ehrman, 1964; Matute, 2010). However, it is also possible that Sfps might, in part, be shaped by selection to reduce the compatibility of interspecific matings, prior to the evolution of complete premating isolation (Billeter & Wolfner, 2018). *D. melanogaster* and *D. yakuba/ D. erecta* are highly diverged and show strong premating barriers, which prevent the occurrence of heterospecific matings (Turissini et al., 2018). However, we found that Acps remained functional and induce strong physiological responses similar to those of conspecifics in these species. This indicates that Acps in these species have not been shaped by selection for mating incompatibilities and that premating barriers in these species evolved rapidly and prior to any divergence in Acp functions. Increasing species divergence is expected to result in degraded interspecific Acp functions over time (Orr, 1996). The finding of a degree of conservation in the re‐mating inhibitory functions between Acps of species as widely diverged as *D. melanogaster* and *D. yakuba*/ *D. erecta* suggests the possibility of evolutionary constraints on at least some Acps and their receptors.
2. *Consequences of sexual conflict in the D. melanogaster species subgroup*. Sfps across a wide variety of taxa evolve rapidly which may be a result of strong or conversely even excessively relaxed selection (Dapper & Wade, 2020; Findlay et al., 2014). In the *D. melanogaster* species subgroup, it has been hypothesized that sexual conflict can promote the rapid evolution of Sfps (Findlay & Swanson, 2010; Hollis et al., 2019; Minekawa et al., 2018; Pitnick et al., 2001; Sirot et al., 2014, 2015). The Sfps of *Drosophila* spp. have multiple functions, but high apparent functional redundancy, which may prevent females from easily evolving resistance to Sfps with manipulative effects (Chapman, 2008, 2018). However, as a side effect, this may also predispose Sfps to retain their ability to effect post‐mating responses in heterospecific females.

It is also possible that the degree of any such redundancy is itself variable across the species tested in this study, which might contribute towards the asymmetric satyrization observed. The production of many different types of Sfps per function is likely to be costly and might also trade off against other traits. For example, *D. sechellia* are endemic to the Seychelles and exhibit relatively low genetic diversity and a small effective population size (David & Capy, 1982; Legrand et al., 2009). *D. simulans* appears to have fewer Sfps than are found in *D. melanogaster* (Findlay et al., 2008). This suggests that either *D. simulans* has shed redundant Sfps or *D. melanogaster* has evolved novel Sfps.

The observed asymmetries suggest that Acps are evolving faster in some lineages than others but that Acp receptors in these rapidly evolving species have broad‐scale specificity. Consequently, these receptors may retain the ability to bind and be activated by less rapidly evolving Acps, resulting in asymmetric effects in reciprocal matings.

## 5 CONCLUSIONS

Here we have found significant asymmetrical satyrization within a single clade of *Drosophila* fruitflies. This work builds upon studies in other Diptera species (Tripet et al., 2011; Turissini et al., 2018; Yassin & David, 2016), to demonstrate that satyrization is present within members of the *D. melanogaster* species subgroup and quantify the pre and post‐mating costs. *Drosophila* exhibit variable premating barriers, with biased heterospecific mating frequency, and significant asymmetries in the post‐mating effects of Acps. This is evidence that asymmetric satyrization is likely much more widespread than has been originally thought and is likely to be an important yet underappreciated factor in speciation, sexual selection and interspecific competition.

## CONFLICT OF INTEREST

The authors declare no conflict of interest.

## AUTHOR CONTRIBUTIONS

TC, SL, LA and MIT devised the experiments, SL conducted the research, collected and analysed the data; SL and TC wrote the paper; all authors contributed to the final draft.

### Peer Review

The peer review history for this article is available at https://publons.com/publon/10.1111/jeb.13733.

## Supporting information

Supplementary Material

## Data Availability

The raw data are deposited in the DRYAD data depository, https://doi.org/10.5061/dryad.0zpc866wc

## References

[jeb13733-bib-0001] Ahmed‐Braimah, Y. H. , Unckless, R. L. , & Clark, A. G. (2017). Evolutionary dynamics of male reproductive genes in the *Drosophila virilis* subgroup. G3: Genes, Genomes, Genetics, 7(9), 3145–3155. 28739599 10.1534/g3.117.1136 PMC5592939

[jeb13733-bib-0002] Alto, B. W. , Smartt, C. T. , Shin, D. , Bettinardi, D. , Malicoate, J. , Anderson, S. L. , & Richards, S. L. (2014). Susceptibility of Florida *Aedes aegypti* and *Aedes albopictus* to dengue viruses from Puerto Rico. Journal of Vector Ecology, 39(2), 406–413. 25424270 10.1111/jvec.12116

[jeb13733-bib-0003] Andrés, J. A. , Maroja, L. S. , & Harrison, R. G. (2008). Searching for candidate speciation genes using a proteomic approach: Seminal proteins in field crickets. Proceedings of the Royal Society B: Biological Sciences, 275(1646), 1975–1983. 10.1098/rspb.2008.0423 PMC2596363 18495616

[jeb13733-bib-0004] Balakrishnan, C. N. , Sefc, K. M. , & Sorenson, M. D. (2009). Incomplete reproductive isolation following host shift in brood parasitic indigobirds. Proceedings of the Royal Society B, 276(1655), 219–228. 18812294 10.1098/rspb.2008.0733 PMC2674339

[jeb13733-bib-0005] Barbash, D. A. (2010). Ninety years of *Drosophila melanogaster* hybrids. Genetics, 186(1), 1–8. 20855573 10.1534/genetics.110.121459 PMC2940278

[jeb13733-bib-0006] Barker, J. S. F. (1962). Sexual isolation between *Drosophila melanogaster* and *Drosophila simulans* . The American Naturalist, 96(887), 105–115.

[jeb13733-bib-0007] Billeter, J. C. , & Wolfner, M. F. (2018). Chemical cues that guide female reproduction in *Drosophila melanogaster* . Journal of Chemical Ecology, 44(9), 750–769. 29557077 10.1007/s10886-018-0947-z PMC6085157

[jeb13733-bib-0008] Castillo, D. M. , & Moyle, L. C. (2019). Conspecific sperm precedence is reinforced, but post copulatory sexual selection weakened, in sympatric populations of *Drosophila* . Proceedings of the Royal Society B, 286, 20182535. 30900533 10.1098/rspb.2018.2535 PMC6452082

[jeb13733-bib-0009] Chapman, T. (2001). Seminal fluid‐mediated fitness traits in *Drosophila* . Heredity, 87(5), 511–521. 11869341 10.1046/j.1365-2540.2001.00961.x

[jeb13733-bib-0010] Chapman, T. (2008). The soup in my fly: Evolution, form and function of seminal fluid proteins. PLoS Biology, 6(7), e179. 18666830 10.1371/journal.pbio.0060179 PMC2486303

[jeb13733-bib-0011] Chapman, T. (2018). Sexual conflict: Mechanisms and emerging themes in resistance biology. The American Naturalist, 192(2), 217–229. 10.1086/698169 30016167

[jeb13733-bib-0012] Chapman, T. , Bangham, J. , Vinti, G. , Seifried, B. , Lung, O. , Wolfner, M. F. , Smith, H. K. , & Partridge, L. (2003). The sex peptide of *Drosophila melanogaster*: Female post‐mating responses analyzed by using RNA interference. Proceedings of the National Academy of Sciences, USA, 100(17), 9923–9928. 10.1073/pnas.1631635100 PMC187888 12893873

[jeb13733-bib-0013] Chapman, T. , & Davies, S. J. (2004). Functions and analysis of the seminal fluid proteins of male *Drosophila melanogaster* fruit flies. Peptides, 25(9), 1477–1490. 15374649 10.1016/j.peptides.2003.10.023

[jeb13733-bib-0014] Coyne, J. A. , & Orr, H. A. (1989). Patterns of speciation in *Drosophila* . Evolution, 43(2), 362–381. 28568554 10.1111/j.1558-5646.1989.tb04233.x

[jeb13733-bib-0015] Coyne, J. A. , & Orr, H. A. (1997). “Patterns of speciation in *Drosophila*” revisited. Evolution, 51(1), 295–303. 28568795 10.1111/j.1558-5646.1997.tb02412.x

[jeb13733-bib-0016] Dapper, A. L. , & Wade, M. J. (2016). The evolution of sperm competition genes: The effect of mating system on levels of genetic variation within and between species. Evolution, 70(2), 502–511. 26748568 10.1111/evo.12848 PMC4868060

[jeb13733-bib-0017] Dapper, A. L. , & Wade, M. J. (2020). Relaxed selection and the rapid evolution of reproductive genes. Trends in Genetics, 36(9), 640–649. 32713599 10.1016/j.tig.2020.06.014

[jeb13733-bib-0018] David, J. R. , & Capy, P. (1982). Genetics and origin of a *Drosophila melanogaster* population recently introduced to the Seychelles. Genetics Research, 40(3), 295–303.

[jeb13733-bib-0019] de Bruyn, P. N. , Tosh, C. A. , & Bester, M. N. (2008). Sexual harassment of a king penguin by an Antarctic fur seal. Journal of Ethology, 26(2), 295–297.

[jeb13733-bib-0020] Ehrman, L. (1964). Courtship and mating behavior as a reproductive isolating mechanism in *Drosophila* . American Zoologist, 4, 147–153. 14172724 10.1093/icb/4.2.147

[jeb13733-bib-0021] Findlay, G. D. , MacCoss, M. J. , & Swanson, W. J. (2009). Proteomic discovery of previously unannotated, rapidly evolving seminal fluid genes in *Drosophila* . Genome Research, 19(5), 886–896. 19411605 10.1101/gr.089391.108 PMC2675977

[jeb13733-bib-0022] Findlay, G. D. , Sitnik, J. L. , Wang, W. , Aquadro, C. F. , Clark, N. L. , & Wolfner, M. F. (2014). Evolutionary rate covariation identifies new members of a protein network required for *Drosophila melanogaster* female post‐mating responses. PLoS Genetics, 10(1), e238. 10.1371/journal.pgen.1004108 PMC3894160 24453993

[jeb13733-bib-0023] Findlay, G. D. , & Swanson, W. J. (2010). Proteomics enhances evolutionary and functional analysis of reproductive proteins. BioEssays, 32(1), 26–36. 20020477 10.1002/bies.200900127

[jeb13733-bib-0024] Findlay, G. D. , Yi, X. , MacCoss, M. J. , & Swanson, W. J. (2008). Proteomics reveals novel *Drosophila* seminal fluid proteins transferred at mating. PLoS Biology, 6(7), e178. 18666829 10.1371/journal.pbio.0060178 PMC2486302

[jeb13733-bib-0025] Goenaga, J. , Yamane, T. , Rönn, J. , & Arnqvist, G. (2015). Within‐species divergence in the seminal fluid proteome and its effect on male and female reproduction in a beetle. BMC Evolutionary Biology, 15(1), 1–13. 26627998 10.1186/s12862-015-0547-2 PMC4667481

[jeb13733-bib-0026] Gröning, J. , & Hochkirch, A. (2008). Reproductive interference between animal species. The Quarterly Review of Biology, 83(3), 257–282. 18792662 10.1086/590510

[jeb13733-bib-0027] Haerty, W. , Jagadeeshan, S. , Kulathinal, R. J. , Wong, A. , Ram, K. R. , Sirot, L. K. , Levesque, L. , Artieri, C. G. , Wolfner, M. F. , Civetta, A. , & Singh, R. S. (2007). Evolution in the fast lane: Rapidly evolving sex‐related genes in *Drosophila* . Genetics, 177(3), 1321–1335. 18039869 10.1534/genetics.107.078865 PMC2147986

[jeb13733-bib-0028] Hollis, B. , Koppik, M. , Wensing, K. U. , Ruhmann, H. , Genzoni, E. , Erkosar, B. , Kawecki, T. J. , Fricke, C. , & Keller, L. (2019). Sexual conflict drives male manipulation of female postmating responses in *Drosophila melanogaster* . Proceedings of the National Academy of Sciences of the United States of America, 116(17), 8437–8444. 30962372 10.1073/pnas.1821386116 PMC6486729

[jeb13733-bib-0029] Hugo, R. L. E. , Stassen, L. , La, J. , Gosden, E. , Winterford, C. , Viennet, E. , Faddy, H. M. , Devine, G. J. , & Frentiu, F. D. (2019). Vector competence of Australian *Aedes aegypti* and *Aedes albopictus* for an epidemic strain of Zika virus. PLoS Neglected Tropical Diseases, 13(4), e0007281. 30946747 10.1371/journal.pntd.0007281 PMC6467424

[jeb13733-bib-0030] Johnson, B. W. , Chambers, T. V. , Crabtree, M. B. , Filippis, A. M. , Vilarinhos, P. T. , Resende, M. C. , Maria de Lourdes, G. M. , & Miller, B. R. (2002). Vector competence of Brazilian *Aedes aegypti* and *Ae. albopictus* for a Brazilian yellow fever virus isolate. Transactions of the Royal Society of Tropical Medicine and Hygiene, 96(6), 611–613. 12625133 10.1016/s0035-9203(02)90326-3

[jeb13733-bib-0031] Kishi, S. , & Nakazawa, T. (2013). Analysis of species coexistence co‐mediated by resource competition and reproductive interference. Population Ecology, 55(2), 305–313.

[jeb13733-bib-0032] Landolt, P. J. , & Heath, R. R. (1987). Role of female‐produced sex pheromone in behavioral reproductive isolation between *Trichoplusia ni* (Hübner) and *Pseudoplusia includens* (Walker) (Lepidoptera: Noctuidae, Plusiinae). Journal of Chemical Ecology, 13(5), 1005–1018. 24302128 10.1007/BF01020534

[jeb13733-bib-0033] Legrand, D. , Tenaillon, M. I. , Matyot, P. , Gerlach, J. , Lachaise, D. , & Cariou, M. L. (2009). Species‐wide genetic variation and demographic history of *Drosophila sechellia*, a species lacking population structure. Genetics, 182(4), 1197–1206. 19506309 10.1534/genetics.108.092080 PMC2728859

[jeb13733-bib-0034] Liu, H. , & Kubli, E. (2003). Sex‐peptide is the molecular basis of the sperm effect in *Drosophila melanogaster* . Proceedings of the National Academy of Sciences of the United States of America, 100(17), 9929–9933. 12897240 10.1073/pnas.1631700100 PMC187889

[jeb13733-bib-0035] Manier, M. K. , Belote, J. M. , Berben, K. S. , Lüpold, S. , Ala‐Honkola, O. , Collins, W. F. , & Pitnick, S. (2013). Rapid diversification of sperm precedence traits and processes among three sibling *Drosophila* species. Evolution, 67(8), 2348–2362. 23888856 10.1111/evo.12117

[jeb13733-bib-0036] Manier, M. K. , Lüpold, S. , Belote, J. M. , Starmer, W. T. , Berben, K. S. , Ala‐Honkola, O. , Collins, W. F. , & Pitnick, S. (2013). Postcopulatory sexual selection generates speciation phenotypes in *Drosophila* . Current Biology, 23(19), 1853–1862. 24076241 10.1016/j.cub.2013.07.086

[jeb13733-bib-0037] Matute, D. R. (2010). Reinforcement of gametic isolation in *Drosophila* . PLoS Biology, 8(3), e1000341. 20351771 10.1371/journal.pbio.1000341 PMC2843595

[jeb13733-bib-0038] Miller, S. E. , Legan, A. W. , Flores, Z. A. , Ng, H. Y. , & Sheehan, M. J. (2019). Strong, but incomplete, mate choice discrimination between two closely related species of paper wasp. Biological Journal of the Linnean Society, 126(3), 614–622. 30853716 10.1093/biolinnean/bly191 PMC6393845

[jeb13733-bib-0039] Minekawa, K. , Miyatake, T. , Ishikawa, Y. , & Matsuo, T. (2018). The adaptive role of a species‐specific courtship behaviour in coping with remating suppression of mated females. Animal Behaviour, 140, 29–37.

[jeb13733-bib-0040] Moulin, B. , Aubin, T. , & Jallon, J. M. (2004). Why there is a one‐way crossability between *D. melanogaster* and *D. simulans*? Genetica, 120(1–3), 285–292. 15088667 10.1023/b:gene.0000017650.45464.f4

[jeb13733-bib-0041] Mueller, J. L. , Ram, K. R. , McGraw, L. A. , Qazi, M. B. , Siggia, E. D. , Clark, A. G. , Aquadro, C. F. , & Wolfner, M. F. (2005). Cross‐species comparison of *Drosophila* male accessory gland protein genes. Genetics, 171(1), 131–143. 10.1534/genetics.105.043844 15944345 PMC1456506

[jeb13733-bib-0042] Noriyuki, S. , Osawa, N. , & Nishida, T. (2012). Asymmetric reproductive interference between specialist and generalist predatory ladybirds. Journal of Animal Ecology, 81(5), 1077–1085. 10.1111/j.1365-2656.2012.01984.x 22537074

[jeb13733-bib-0043] Obbard, D. J. , Maclennan, J. , Kim, K. W. , Rambaut, A. , O’Grady, P. M. , & Jiggins, F. M. (2012). Estimating divergence dates and substitution rates in the *Drosophila* phylogeny. Molecular Biology and Evolution, 29(11), 3459–3473. 10.1093/molbev/mss150 22683811 PMC3472498

[jeb13733-bib-0044] Orr, H. A. (1996). Dobzhansky, Bateson, and the genetics of speciation. Genetics, 144(4), 1331. 8978022 10.1093/genetics/144.4.1331 PMC1207686

[jeb13733-bib-0045] Pavković‐Lučić, S. , Lučić, L. , Miličić, D. , Tomić, V. , & Savić, T. (2014). Mating success and copulation duration in *Drosophila melanogaster* flies having different mating experience: A brief experimental note. Journal of BioScience & Biotechnology 2014, 153–159.

[jeb13733-bib-0046] Pitnick, S. , Brown, W. D. , & Miller, G. T. (2001). Evolution of female remating behaviour following experimental removal of sexual selection. Proceedings of the Royal Society B, 268(1467), 557–563. 11297171 10.1098/rspb.2000.1400 PMC1088640

[jeb13733-bib-0047] Price, C. S. (1997). Conspecific sperm precedence in *Drosophila* . Nature, 388(6643), 663–666. 9262398 10.1038/41753

[jeb13733-bib-0048] R Core Team . (2012). R: A language and environment for statistical computing. R Foundation for Statistical Computing.

[jeb13733-bib-0049] Ribeiro, J. M. C. , & Spielman, A. (1986). The satyr effect: A model predicting parapatry and species extinction. The American Naturalist, 128(4), 513–528. 10.1086/284584

[jeb13733-bib-0050] Rubinstein, C. D. , & Wolfner, M. F. (2013). Drosophila seminal protein ovulin mediates ovulation through female octopamine neuronal signaling. Proceedings of the National Academy of Sciences, USA, 110(43), 17420–17425. 10.1073/pnas.1220018110 PMC3808635 24101486

[jeb13733-bib-0051] Sato, Y. , Breeuwer, J. A. , Egas, M. , & Sabelis, M. W. (2015). Incomplete premating and postmating reproductive barriers between two parapatric populations of a social spider mite. Experimental and Applied Acarology, 65(3), 277–291. 25633263 10.1007/s10493-015-9878-3 PMC4322225

[jeb13733-bib-0052] Schwarz, D. , & McPheron, B. A. (2007). When ecological isolation breaks down: Sexual isolation is an incomplete barrier to hybridization between *Rhagoletis* species. Evolutionary Ecology Research, 9(5), 829–841.

[jeb13733-bib-0053] Seehausen, O. , Van Alphen, J. J. , & Witte, F. (1997). Cichlid fish diversity threatened by eutrophication that curbs sexual selection. Science, 277(5333), 1808–1811.

[jeb13733-bib-0054] Sepil, I. , Hopkins, B. R. , Dean, R. , Thézénas, M. L. , Charles, P. D. , Konietzny, R. , Fischer, R. , Kessler, B. M. , & Wigby, S. (2019). Quantitative proteomics identification of seminal fluid proteins in male *Drosophila melanogaster* . Molecular & Cellular Proteomics, 18(Suppl. 1), S46–S58. 30287546 10.1074/mcp.RA118.000831 PMC6427238

[jeb13733-bib-0055] Shuker, D. M. , & Burdfield‐Steel, E. R. (2017). Reproductive interference in insects. Ecological Entomology 42, 65–75.

[jeb13733-bib-0056] Sirot, L. K. , Buehner, N. A. , Fiumera, A. C. , & Wolfner, M. F. (2009). Seminal fluid protein depletion and replenishment in the fruit fly, *Drosophila melanogaster*: An ELISA‐based method for tracking individual ejaculates. Behavioral Ecology and Sociobiology, 63(10), 1505–1513. 24733957 10.1007/s00265-009-0806-6 PMC3984576

[jeb13733-bib-0057] Sirot, L. K. , Findlay, G. D. , Sitnik, J. L. , Frasheri, D. , Avila, F. W. , & Wolfner, M. F. (2014). Molecular characterization and evolution of a gene family encoding both female‐and male‐specific reproductive proteins in *Drosophila* . Molecular Biology and Evolution, 31(6), 1554–1567. 24682282 10.1093/molbev/msu114 PMC4032134

[jeb13733-bib-0058] Sirot, L. K. , LaFlamme, B. A. , Sitnik, J. L. , Rubinstein, C. D. , Avila, F. W. , Chow, C. Y. , & Wolfner, M. F. (2009). Molecular social interactions: Drosophila melanogaster seminal fluid proteins as a case study. In Advances in genetics (Vol. 68, pp. 23–56). Academic Press. 20109658 10.1016/S0065-2660(09)68002-0 PMC3925388

[jeb13733-bib-0059] Sirot, L. K. , Wong, A. , Chapman, T. , & Wolfner, M. F. (2015). Sexual conflict and seminal fluid proteins: A dynamic landscape of sexual interactions. Cold Spring Harbor Perspectives in Biology, 7(2), a017533. 10.1101/cshperspect.a017533 PMC4315932 25502515

[jeb13733-bib-0060] Sperlich, D. (1962). Hybrids between *Drosophila melanogaster* and *D. simulans* in nature. Drosophila Information Service, 36, 118.

[jeb13733-bib-0061] Sturtevant, A. H. (1920). Genetic studies on *Drosophila simulans*. I. Introduction. Hybrids with *Drosophila melanogaster* . Genetics, 5(5), 488. 17245951 10.1093/genetics/5.5.488 PMC1200491

[jeb13733-bib-0062] Swanson, W. J. , & Vacquier, V. D. (2002). The rapid evolution of reproductive proteins. Nature Reviews Genetics, 3(2), 137. 10.1038/nrg733 11836507

[jeb13733-bib-0063] Tripet, F. , Lounibos, L. P. , Robbins, D. , Moran, J. , Nishimura, N. , & Blosser, E. M. (2011). Competitive reduction by satyrization? Evidence for interspecific mating in nature and asymmetric reproductive competition between invasive mosquito vectors. The American Journal of Tropical Medicine and Hygiene, 85(2), 265–270. 10.4269/ajtmh.2011.10-0677 21813845 PMC3144823

[jeb13733-bib-0064] Tsuda, M. , & Aigaki, T. (2016). Evolution of sex‐peptide in Drosophila. Fly, 10(4), 172–177. 10.1080/19336934.2016.1193655 27230918 PMC5036924

[jeb13733-bib-0065] Turissini, D. A. , McGirr, J. A. , Patel, S. S. , David, J. R. , & Matute, D. R. (2018). The rate of evolution of postmating‐prezygotic reproductive isolation in *Drosophila* . Molecular Biology and Evolution, 35(2), 312–334. 10.1093/molbev/msx271 29048573 PMC5850467

[jeb13733-bib-0066] van Doorn, G. S. , Edelaar, P. , & Weissing, F. J. (2009). On the origin of species by natural and sexual selection. Science, 326(5960), 1704–1707. 19965377 10.1126/science.1181661

[jeb13733-bib-0067] Yassin, A. , & David, J. R. (2016). Within‐species reproductive costs affect the asymmetry of satyrization in *Drosophila* . Journal of Evolutionary Biology, 29(2), 455–460. 26538290 10.1111/jeb.12784

