# Supplementary material for: Satyrization in Drosophila fruitflies
Source: J Evol Biol. 2020 Dec 2;34(2):319–30. doi: 10.1111/jeb.13733 (PMC8246970; doi:10.1111/jeb.13733)
Supplement: Supplementary file 1 — Supplementary Material [file JEB-34-319-s001.pdf]

Satyrization in *Drosophila* fruiflies

Stewart Leigh[1], Wayne G. Rostant[1], Martin I. Taylor[1], Luke Alphey[2] and Tracey Chapman[1*]

[1]School of Biological Sciences, University of East Anglia, Norwich Research Park, Norwich, NR4 7TJ, UK.

[2]The Pirbright Institute, Ash Road, Woking, GU24 0NF, UK.

***Corresponding author:** Tracey Chapman, tracey.chapman@uea.ac.uk

Supplementary Information

Supplementary figures:

Figure S1: Experimental set up of the initial heterospecific mating experiments (experiments A, B)

Figure S2: Experimental set up of the Acp injection experiments (experiment C)

Supplementary tables:

Table S1: Number of females set up and mated in heterospecific and conspecific first matings followed by conspecific second matings between *D. melanogaster* and *D. simulans*

Table S2: Survival data following Acp injection into *Drosophila* females

Supplementary results:

1. Detailed statistical results for individual pair comparisons for reciprocal Acp receipt across the *D. melanogaster* species subgroup
2. Detailed Statistical analysis of variable mortality to Acp injections

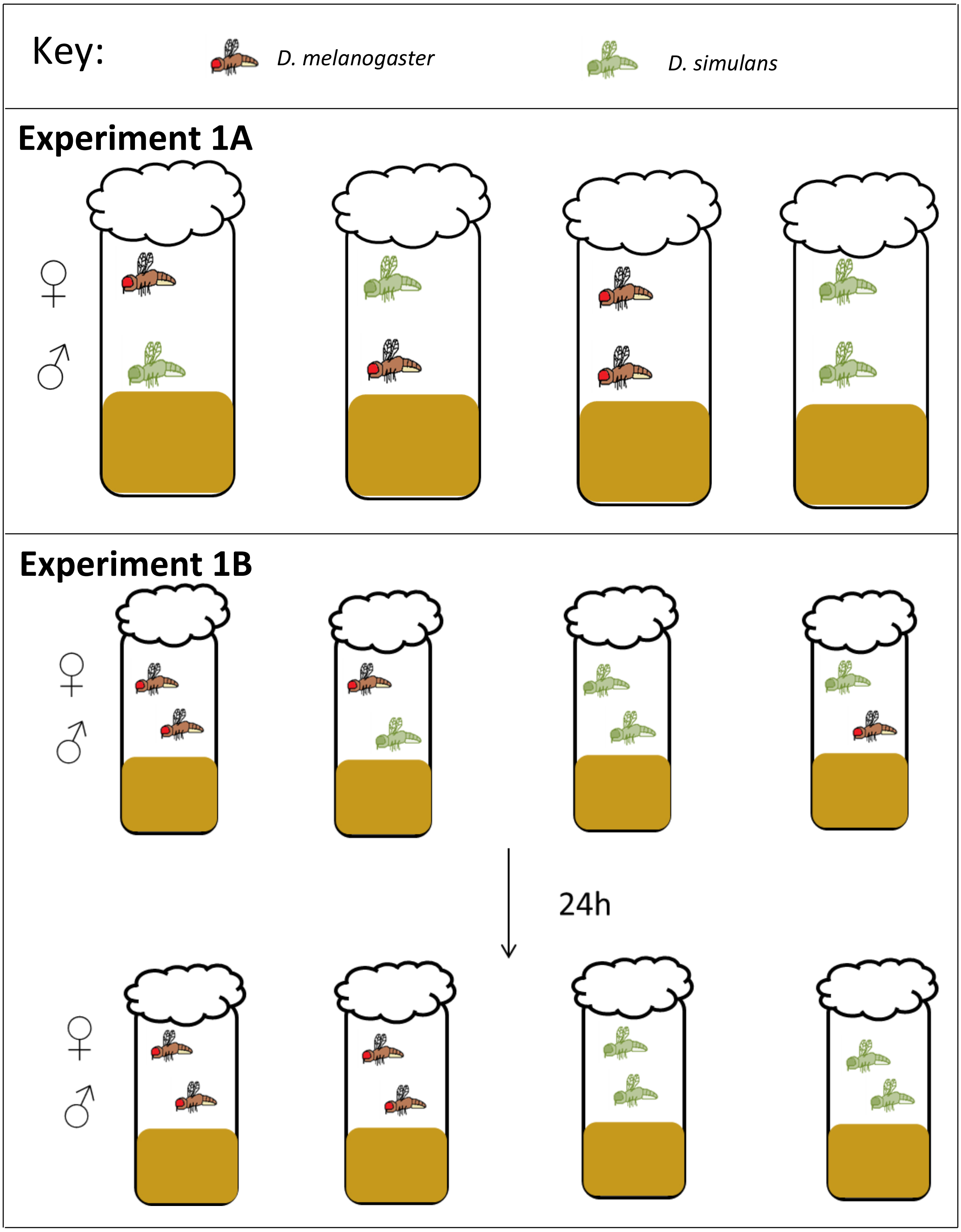

Figure S1: Experimental set up of the initial heterospecific mating experiments (Experiments 1A, 1B, main text). In the first experiment (**Experiment 1A**) *D. melanogaster* and *D. simulans* were set up to mate in conspecific and heterospecific pairs for 3h. Pairs were continuously watched and mating latency was recorded. In the second experiment (**Experiment 1B**) *D. melanogaster* and *D. simulans* were again set up in conspecific and heterospecific pairs for 3h. Mating latency was recorded and mated females were retained. After 24h, all previously mated females were given the opportunity to remate with a conspecific male. Pairs were again observed continuously for 3h and mating latency recorded.

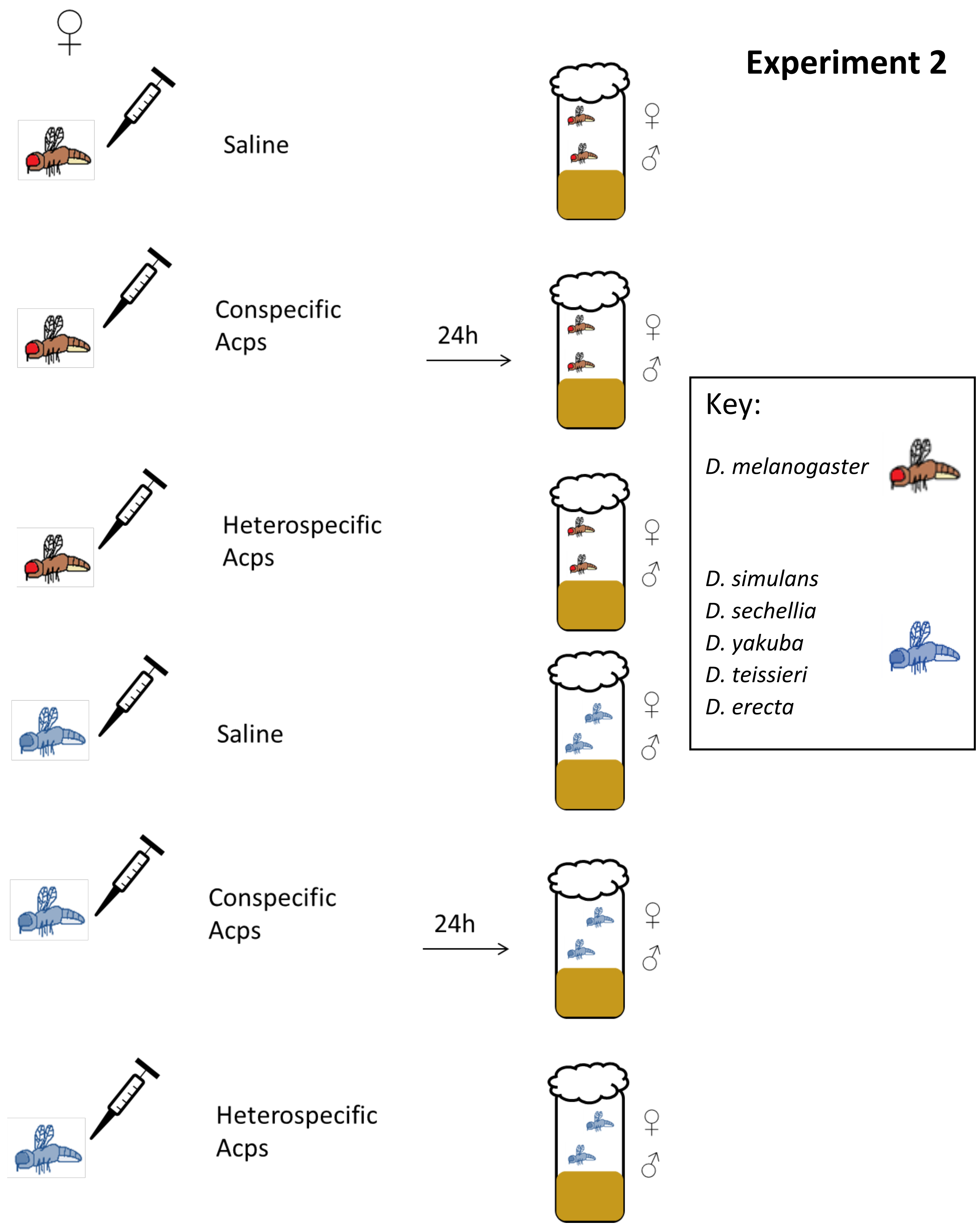

Figure S2: Experimental set up of the Acp injection experiments (Experiment 2, main text). Each experiment consisted of *D. melanogaster* and one other member of the *D. melanogaster* species subgroup (*D. simulans*, *D. sechellia*, *D. yakuba*, *D. teissieri* and *D. erecta*). Females from each species were injected with 0.1µl of either 1xPBS Saline, conspecific Acps or heterospecific Acps from the reciprocal species in each experiment. After 24h, females were given the opportunity to mate with conspecific males. Pairs were observed continuously for 3h during which mating latency was recorded.

Table S1: Number of females set up and mated in heterospecific and conspecific first matings followed by conspecific second matings, in *D. melanogaster* and *D. simulans*. Females in each treatment were mated twice. In the first mating, females were set up in pairs with either a conspecific or heterospecific male. In the second mating, mated females from the first mating were set up in pairs with a conspecific male. The number of pairs set up and the number and percentage of pairs that mated in each mating are shown.

First Mating			Second Mating		
	Number of Pairs Set Up	Number and Percentage of Pairs that Mated		Number of Pairs Set Up	Number and Percentage of Pairs that Mated
D. melanogaster (♀) x *D. melanogaster* (♂)	**52**	**48** *(92.3%)*	*D. melanogaster* (♀) x *D. melanogaster* (♂)	**48**	**17** *(37%)*
D. simulans (♀) x *D. simulans* (♂)	**30**	**27** *(93.3%)*	*D. simulans* (♀) x *D. simulans* (♂)	**25**	**3** *(12%)*
D. simulans (♀) x *D. melanogaster* (♂)	**136**	**47** *(34.6%)*	*D. simulans* (♀) x *D. simulans* (♂)	**31**	**11** *(39.3%)*
D. melanogaster (♀) x *D. simulans* (♂)	**82**	**0** *(0%)*	*D. melanogaster* (♀) x *D. melanogaster* (♂)	**0**	**n/a**

Table S2: Survival data following Acp injection into *Drosophila* females. Females of each species were initially injected with either conspecific Acps, heterospecific Acps, or saline. The number of surviving females 24h post-injection and total number of injected females are shown in bold, the percentage of surviving females 24h post injection are stated in italics.

		**Number of (Surviving/Total Injected) Females 24h Post-Injection** *(% of Surviving Females 24h Post-Injection)*		
		Saline	Conspecific Acps	Heterospecific Acps
Species pairs used in each experiment	*D. yakuba*	**(55/77)** *(71.4%)*	**(30/80)** *(37.5%)*	**(18/79)** *(22.8%)*
	D. melanogaster	**(71/80)** *(88.8%)*	**(66/78)** *(84.6%)*	**(64/79)** *(81%)*
	D. erecta	**(72/80)** *(90%)*	**(65/80)** *(81.3%)*	**(40/80)** *(50%)*
	D. melanogaster	**(72/80)** *(90%)*	**(63/80)** *(78.8%)*	**(62/70)** *(88.6%)*
	D. simulans	**(57/85)** *(67.1%)*	**(68/84)** *(81%)*	**(58/75)** *(77.3%)*
	D. melanogaster	**(70/80)** *(87.5%)*	**(45/80)** *(56.3%)*	**(36/80)** *(45%)*
	D. sechellia	**(63/80)** *(78.8%)*	**(25/74)** *(33.8%)*	**(58/75)** *(77.3%)*
	D. melanogaster	**(74/80)** *(92.5%)*	**(71/80)** *(88.8%)*	**(74/80)** *(92.5%)*
	D. teissieri	**(59/80)** *(73.8%)*	**(36/80)** *(45%)*	**(33/80)** *(41.3%)*
	D. melanogaster	**(70/80)** *(87.5%)*	**(66/80)** *(82.5%)*	**(65/80)** *(81.3%)*

Supplementary results

1. Detailed statistical results for individual pair comparisons for reciprocal Acp receipt across the *D. melanogaster* species subgroup

D. melanogaster* x *D. simulans: *D. simulans* females showed significantly reduced receptivity from heterospecific *D. melanogaster* Acps (Hazard Ratio (HR): 0.13; 95% Confidence Intervals (CI) [0.06, 0.3]; P=9.7e-07) and conspecific *D. simulans* Acps (HR: 0.32; 95% CI [0.18, 0.55]; P=4.7e-05), in comparison to the saline control (figure 3A). However, *D. melanogaster* Acps reduced receptivity in *D. simulans* females to a greater extent than did conspecific *D. simulans* Acps (HR: 0.41; 95% CI [0.17, 0.97]; P=0.043). Conspecific *D. melanogaster* Acps also significantly reduced *D. melanogaster* female receptivity (HR: 0.53; 95% CI [0.35, 0.8]; P=0.002) compared to the saline control, but *D. simulans* Acps had no such effect (HR: 0.78; 95% CI [0.51, 1.2]; P=0.262). Collectively, there was evidence for significant asymmetry, with *D. melanogaster* Acps having a significant inhibitory effect on receptivity in females of both species, but with *D. simulans* Acps affecting only conspecific receptivity. The GLM showed a significant interaction effect between the injection treatments and the species of injected female ($F_{(2,312)}$=4.742; P=0.009. Saline (z=3.880, P=0.0001), *D. melanogaster* Acps (z=6.752; P<0.0001) and *D. simulans* Acps (z=6.977; P<0.0001) had a significantly different post-mating effects in *D. melanogaster* vs *D. simulans* females.

D. melanogaster* x *D. sechellia: *D. melanogaster* female receptivity was significantly reduced by both *D. sechellia* Acps (HR: 0.35; 95% CI [0.25, 0.5]; P=3.8e-09) and *D. melanogaster* Acps (HR: 0.07; 95% CI [0.04, 0.11]; P<2e-16) compared to the saline control. However, *D. sechellia* Acps induced a significantly weaker refractory response in *D. melanogaster* females than was found for conspecific Acps (HR: 0.2; 95% CI [0.13, 0.32]; P=4.58e-12) demonstrating an asymmetrical effect acting on the ability of Acps to reduce mating receptivity (figure 3B). In contrast, *D. sechellia* females showed an equivalent significant reduction in receptivity upon receipt of Acps from *D. melanogaster* (HR: 0.02; 95% CI [0.003, 0.13]; P=7.1e-05) and *D. sechellia* (HR: 0.08; 95% CI [0.02, 0.3]; P=6.2e-04) compared to the saline control. The GLM showed a significant interaction effect between the injection treatment and the species of injected female ($F_{(2,361)}$=15.825; P=2.6e-07). Saline (z=9.223, P<0.0001), *D. melanogaster* Acps (z=3.967; P=0.0001) and *D. sechellia* Sfps (z=9.924; P<0.0001) had a significantly different post-mating effect in *D. melanogaster* vs *D. sechellia* females.

D. melanogaster* x *D. teissieri: *D. melanogaster* females showed significantly reduced mating receptivity following receipt of conspecific Acps (HR: 0.29; 95% CI [0.19, 0.44]; P=6.51e-09) but not heterospecific *D. teissieri* Acps (HR: 0.82; 95% CI [0.56, 1.2]; P=0.307) compared to the saline control.

However, *D. teissieri* females exhibited an equivalent significant reduction in receptivity upon receipt of *D. melanogaster* Acps (HR: 0.14; 95% CI [0.05, 0.35]; P=3.37e-05) and *D. teissieri* Sfps (HR: 0.21; 95% CI [0.1, 0.46]; P=6.90e-05) compared to the saline control (figure 3C). Thus, as found in *D. sechellia* and *D. simulans*, there was a significant asymmetry in female post-mating receptivity responses. The GLM showed significant interaction effects between the injection treatment and the species of injected female ($F_{(2,316)}$=7.31; P=7.89e-04). Saline (z=4.28, P<0.0001), *D. melanogaster* Acps (z=3.51; P=0.0004) and *D. teissieri* Sfps (z=8.15; P<0.0001) had a significantly different post-mating effect in *D. melanogaster* vs *D. teissieri* females.

D. melanogaster* x *D. erecta: *D. erecta* females showed significantly reduced receptivity following receipt of conspecific *D. erecta* (HR: 0.33; 95% CI [0.16, 0.67]; P=2.21e-03) and heterospecific *D. melanogaster* Acps (HR: 0.1; 95% CI [0.02, 0.41]; P=1.51e-03) compared to the saline control. *D. melanogaster* females showed similarly significantly reduced mating receptivity following receipt of *D. erecta* (HR: 0.2; 95% CI [0.13, 0.32]; P=1.64e-12) and *D. melanogaster* Acps (HR: 0.18; 95% CI [0.12, 0.29]; P=2.26e-13) (figure 4A). In this case Acps caused equally effective reductions in receptivity in females of the other species and there was no asymmetry of Acp effects. The GLM showed significant interaction effects between the injection treatment and the species of injected female ($F_{(2,359)}$=8.99; P=1.546e-04). Saline (z=9.45, P<0.0001), *D. melanogaster* Acps (z=3.90; P=0.0001) and *D. erecta* Acps (z=3.46; P=0.0005) had a significantly different post-mating effect in *D. melanogaster* vs *D. erecta* females.

D. melanogaster* x *D. yakuba: *D. melanogaster* female mating receptivity was significantly reduced by receipt of *D. melanogaster* (HR: 0.23; 95% CI [0.15, 0.35]; P=1.48e-11) and *D. yakuba* Sfps (HR: 0.16; 95% CI [0.1, 0.26]; P=1.50e-14) compared to the saline control. Similarly, *D. yakuba* receptivity was significantly reduced by both *D. melanogaster* (HR: 0.22; 95% CI [0.09, 0.52]; P=5.47e-04) and *D. yakuba* Sfps (HR: 0.15; 95% CI [0.07, 0.34]; P=3.76e-06; figure 4B). Hence as for the tests above with *D. erecta*, Acps induced a similar post-mating effect profile within and across species and there was no evidence for asymmetry. The GLM showed no significant interactions between the effect of the injection treatment and the species of the injected female ($F_{(2,298)}$=0.2; P=0.816)

2. Detailed Statistical analysis of variable mortality to Acp injections

D. melanogaster: *D. melanogaster* females showed no significant differences in mortality rate between injections of saline, conspecific or heterospecific Acps in almost all experiments (*D. melanogaster* x *D. sechellia* - χ^2_2=0.94; P=0.625. *D. melanogaster* x *D. teissieri* - χ^2_2=1.29; P=0.526. *D. melanogaster* x *D. erecta* - χ^2_2=4.82; P=0.09. *D. melanogaster* x *D. yakuba* - χ^2_2=1.85; P=0.396). However, in *D. melanogaster* x *D. simulans* there was a significant dfference in *D. melanogaster* female mortality between treatments (χ^2_2=33.25; P=6.016e-08) with saline injected females suffering lower mortality than the conspecific (P=2.443e-05) and heterospecific (P=3.443e-08) Acp-injected females (Table S2). ***D. simulans***: *D. simulans* showed no variability in mortality rate from injections of saline, conspecific or heterospecific Acps (χ^2_2=4.66; P=0.097) (Table S2). ***D. sechellia***: *D. sechellia* females suffered significantly higher mortality from conspecific Acp injection compared to heterospecific Acps and the saline control (χ^2_2=42.53; P=5.804e-10) (Table S2). ***D. teissieri***: There was higher mortality in *D. teissieri* injected with conspecific and heterospecific Acps compared to the saline control (χ^2_2=20.32; P=3.862e-05) (Table S2). ***D. erecta***: *D. erecta* females suffered significantly higher mortality from heterospecific Acp injections compared to conspecific Acp injections and the saline control (χ^2_2=36.55; P=1.159e-08) (Table S2). ***D. yakuba***: *D. yakuba* females suffered significantly higher mortality from both conspecific and heterospecific Acp injections compared to the saline control (χ^2_2=39.37; P=2.824e-9) (Table S2).
